# Antimicrobial and Antiproliferative Activity of Green Synthesized Silver Nanoparticles Using Bee Bread Extracts

**DOI:** 10.3390/pharmaceutics15071797

**Published:** 2023-06-23

**Authors:** Adriana Cristina Urcan, Adriana Dalila Criste, Karina Ioana Szanto, Razvan Ștefan, Marius Zahan, Adriana Sebastiana Muscă, Monica Focsan, Ramona Flavia Burtescu, Neli Kinga Olah

**Affiliations:** 1 Faculty of Animal Science and Biotechnologies, University of Agricultural Sciences and Veterinary Medicine, 3-5 Mănăştur Street, 400372 Cluj-Napoca, Romania; adriana.criste@usamvcluj.ro (A.D.C.); karina_szanto06@yahoo.com (K.I.S.); mzahan@usamvcluj.ro (M.Z.); muscaadrianas@gmail.com (A.S.M.); 2 Faculty of Veterinary Medicine, University of Agricultural Sciences and Veterinary Medicine, 400372 Cluj-Napoca, Romania; rstefan@usamvcluj.ro; 3 Nanobiophotonics and Laser Microspectroscopy Center, Interdisciplinary Research Institute in Bio-Nano-Sciences, Babes-Bolyai University, Treboniu Laurian No. 42, 400271 Cluj-Napoca, Romania; monica.iosin@ubbcluj.ro; 4 PlantExtrakt Ltd., Rădaia, 407059 Cluj-Napoca, Romania; ramona.burtescu@plantextrakt.ro (R.F.B.); neliolah@yahoo.com (N.K.O.); 5 Faculty of Pharmacy, “Vasile Goldiş” Western University of Arad, 310414 Arad, Romania

**Keywords:** green synthesis, bee bread, silver nanoparticles, bioactive compounds, antimicrobial activity, colon cancer

## Abstract

Bee bread (BB) is a fermented mixture of bee pollen, is rich in proteins, amino acids, fatty acids, polyphenols, flavonoids, as well as other bioactive compounds, and is considered functional food for humans. In this study, we explored an innovative green synthesis of colloidal silver nanoparticles, using BB extracts as reducing and stabilizing agents. A preliminary chemical characterization of the BB extracts was conducted. The plasmonic response of the as-synthesized silver nanoparticles (BB-AgNPs) was evaluated by UV–Vis spectroscopy, while their hydrodynamic diameter and zeta potential were investigated by dynamic light spectroscopy (DLS). Transmission electron microscopy (TEM) analysis pointed out polydisperse NPs with quasi-spherical shapes. The newly synthesized nanoparticles showed good antioxidant activity against the tested free radicals, DPPH, ABTS^•+^, and FRAP, the best results being obtained in the case of ABTS^•+^. BB-AgNPs exhibited good antibacterial activity on the tested Gram-positive and Gram-negative bacterial strains: herein *S. aureus*, *B. cereus*, *E. faecalis*, *E. coli*, *P. aeruginosa*, *S. enteritidis*, and on yeast *C. albicans*, respectively. The inhibition diameters varied between 7.67 ± 0.59 and 22.21 ± 1.06 mm, while the values obtained for minimum inhibitory concentration varied between 0.39 and 6.25 µg/mL. In vitro antiproliferative activity was tested on colon adenocarcinoma, ATCC HTB-37 cell line, and the results have shown that the green synthetized BB-AgNPs induced a substantial decrease in tumor cell viability in a dose-dependent manner with an IC50 ranging from 24.58 to 67.91 µg/mL. Consequently, more investigation is required to comprehend the processes of the cytotoxicity of AgNPs and develop strategies to mitigate their potentially harmful effects while harnessing their antimicrobial properties.

## 1. Introduction

Nowadays, nanotechnology has gained great attention as a science field for the design of different nanomaterials, which have direct applicability in different areas such as biomedicine, pharmaceutics, drug delivery, biosensing, diagnostic, bioimaging, catalysis, cosmetology, agriculture, environmental engineering, or food technology because of their excellent biocompatibility, rich surface chemistry, rapid production, and cost-effectiveness [1].

Silver nanoparticles (AgNPs), in particular, have attracted significant attention in a multitude of scientific fields due to their unique physical, chemical, and biological properties. AgNPs are commonly synthesized by various methods, including chemical, physical, and biological routes [2]. Green synthesis methods of AgNPs have gained popularity in recent years because it uses natural, renewable, and environmentally friendly sources for the synthesis of nanoparticles, rather than traditional chemical methods that often involve the use of toxic and hazardous chemicals, which can hurt both human health and the environment [3,4].

In general, green synthesis of AgNPs can be achieved using various extracts, including those from plants’ leaves, stems, flowers, roots, pollen, or microorganisms metabolites [2]. These extracts contain various phytochemicals, including flavonoids, phenolic acids, terpenoids, alkaloids, saccharides, and vitamins, as well as different enzymes, amino acids, and proteins [5,6] that can act as reducing and stabilizing agents for the synthesis of AgNPs [7]. Most of the existing studies in the literature show that different plant extracts and microorganisms [4,8,9,10,11] were used for the green synthesis of silver nanoparticles and reported that the obtained AgNPs exhibit antioxidant activity, antimicrobial activity, or anticancer activity. On the other hand, only a few studies were found that investigated the green synthesis of AgNPs using bee pollen [12,13], and the authors showed that the obtained AgNPs had antioxidant activity, antimicrobial, and anticancer activities.

AgNPs’ distinctive physicochemical characteristics, such as their small size, high surface area, and surface chemistry, are thought to be responsible for their antioxidant activity. These properties enable AgNPs to interact with free radicals and other reactive oxygen species (ROS) and neutralize their negative effects by donating electrons or stabilizing them with hydrogen atoms [14,15].

The resistance of microorganisms to antibiotics is a growing concern in the field of global health, which increasingly compromises the effectiveness of many conventional antibiotics. The misuse and overuse of antibiotics have accelerated the development of antibiotic resistance in many bacterial strains, making it difficult to treat infections with conventional antibiotics [16]. As a result, there is an urgent need for alternative treatments that can inhibit antibiotic-resistant bacteria. One potential alternative to conventional antibiotics can be the use of AgNPs, which showed enhanced antimicrobial activity against a wide range of bacteria, including drug-resistant strains, an improved biocompatibility, and cost efficiency [17]. The high surface area-to-volume ratio of AgNPs enables them to interact effectively with biological targets, such as bacteria and viruses, leading to their destruction [18]. In addition, AgNPs often have multiple modes of action, such as disrupting the bacterial cell membrane, enzyme inactivation, and inhibition of DNA replication, making it difficult for microorganisms to develop resistance [19].

Furthermore, in the biomedical field, besides the antibacterial effect, AgNPs obtained by green synthesis have shown excellent antifungal and antiviral properties [20]. AgNPs have also been reported to have anti-inflammatory and wound-healing properties, making them a potential candidate for various therapeutic applications, including tissue engineering and cancer treatment [21].

Despite the many advantages of AgNPs, there are also some concerns regarding their safety. For example, the small size of AgNPs makes it possible for them to penetrate cells and tissues, leading to potential toxicity, especially at high concentrations. The size of AgNPs is an essential parameter that can influence their therapeutic efficacy, pharmacokinetic, and biodistribution. For instance, small AgNPs have been shown to have a higher cellular uptake and more prolonged circulation time than larger AgNPs, making them a potential candidate for drug delivery and cancer therapy [22,23]. The size of AgNPs also plays a crucial role in their toxicity and biocompatibility. Small AgNPs, generally below 50 nm [24,25], are more toxic to cells and have a higher potential to penetrate biological barriers, such as cell membranes and the blood–brain barrier. In contrast, larger AgNPs have been found to be less toxic and more biocompatible [26].

Bee products such as propolis, bee bread, pollen, honey, royal jelly, or bee venom are regarded as promising sources of antioxidants as they are rich in bioactive compounds such as flavonoids (e.g., kaempferol, quercetin, naringenin) as well as phenolic compounds, including derivatives of cinnamic and benzoic acid [27,28,29,30]. The therapeutic impact of bee products has been predominantly associated with the plant species and geographic origin of their beehives [13,31].

BB is a mixture of bee pollen, digestive enzymes, and honey that has been stored and fermented by worker bees in honeycomb cells. It has a high nutritional value and includes multiple bioactive compounds, which have potential health benefits for humans and is therefore regarded as a functional food. BB is a fully balanced source of protein containing all the essential amino acids, omega fatty acids, simple sugars, and other phytochemicals. It is similar to bee pollen and their composition varies by botanical origin [32,33,34,35,36,37,38]. BB is rich in bioactive substances such as flavonoids, phenolic acids, bioactive peptides, carotenoids, organic acids, vitamins, especially B group vitamins such as thiamine, riboflavin, biotin, nicotinic and folic acid, E, K, C and micro- and macro-elements, such as Ca, Fe, P, K, Zn, Cu, Se, and Mg [39,40,41]. It also contains enzymes (amylase, saccharase, phosphatases) and probiotic bacteria that can aid in digestion and boost the immune system. Some studies suggest that BB may have anti-inflammatory and antimicrobial properties, as well as potential benefits for cardiovascular health or hepatoprotective effect [42,43]. Due to the abundance of bioactive compounds in the composition of BB—within polyphenols and flavonoids represent the most significant group of secondary compounds—numerous studies have been carried out to demonstrate the beneficial effects of this bee product, such as antioxidant, antimicrobial, antitumoral, and anti-inflammatory effects or against drug-induced toxicities both in vitro and in vivo [36,44,45,46,47,48].

Considering all of this, BB is a promising source of bioactive molecules that can be used in the green synthesis of AgNPs.

To the best of our knowledge, BB has not been utilized for the green synthesis of AgNPs so far. In this context, the main aim of this study consisted of green synthesis and the structural characterization of silver nanoparticles (AgNPs) using aqueous extracts of bee bread, as well as the preliminary assessment of the antioxidant, antibacterial, and antiproliferative (colon adenocarcinoma, ATCC HTB-37 cell line) potential of the obtained AgNPs.

## 2. Materials and Methods

### 2.1. Chemicals and Reagents

All chemicals were of analytical grade purity and were obtained from Sigma-Aldrich and Merck (Merck KGaA, Darmstadt, Germany, and/or its affiliates). Milli-Q water pH = 2.4 (with o-phosphoric acid) was used for standard and mobile phase preparation. All reagents and sample extracts were filtered through a 0.45 μm MF-Millipore™ Membrane Filter from Merck (Darmstadt, Germany). Ultrapure water was prepared by Simplicity Ultrapure Water Purification System (Merck Millipore, Billerica, MA, USA).

### 2.2. Samples Collection and Preparation of Bee Bread Extracts

BB samples were collected from *Apis mellifera* L. hives located in the north–west and central counties of Transylvania, Romania. To maintain BB quality, samples were stored in a freezer (−18 °C) until they were analyzed. The botanical origin of the BB sample was identified at the University of Agricultural Science and Veterinary Medicine. The identification methodology was outlined in a prior study performed by our research team [32], and the results showed that all six analyzed samples had different botanical origins.

An amount of 1 g of each BB sample was extracted with 5 mL of ultrapure water in an ultrasonic bath at 30 °C for 60 min (Bandelin Sonorex, model Sonorex Super RK 100 H, Bandelin Electronic GmbH and Co. KG). After sonication, the samples ware centrifuged (15,269× *g*) for 10 min and the supernatants were collected and preserved for further analysis (−4 °C).

### 2.3. Characterization of BB Extracts

#### 2.3.1. Total Phenolic Content (TPC)

The Folin–Ciocalteu method [49] was conducted for total phenolic determination. Briefly, 10 µL of each BB extract was added to 100 µL Folin–Ciocalteu reagent, which was previously diluted (1:10 with de-ionized water; 0.2 M—with respect to acid). Subsequently, 80 µL sodium carbonate (Na_2_CO_3_) solution (1 M) was added; the mixture was left to react for 15 min and then the absorbance was read at 765 nm. Quantification was performed based on a calibration curve of a gallic acid solution whose concentration varied between 0.025 and 0.15 mg/mL (R^2^ = 0.9992). The obtained values were reported as mg of GAE (gallic acid equivalent) per g of dry matter sample. The experiments were conducted in triplicate, utilizing a 96-well microplate reader (Synergy™ HT BioTek Instruments, Winooski, VT, USA).

#### 2.3.2. Total Flavonoid Content (TFC)

Total flavonoid content was determined spectrophotometrically using the method of Mărghitaș et al. [50]. Each tested sample (25 µL) was combined with 100 µL of ultrapure water, followed by the addition of 10 µL of a 5% sodium nitrate (NaNO_2_) solution. After 5 min of incubation, 15 µL of a 2% aluminum chloride (AlCl_3_) solution was added. The mixture was supplemented with 50 µL of 1 M sodium hydroxide (NaOH) and an additional 50 µL of ultrapure water after another 6 min,. The absorbance was measured at 510 nm. Quantification was performed based on a calibration curve of a quercetin solution whose concentration varied between 0.025 and 0.2 mg/mL (R^2^ = 0.9987). The obtained values were reported as mg of Qe (quercetin equivalent) per g of dry matter sample. The experiments were conducted in triplicate utilizing a 96-well microplate reader (Synergy™ HT BioTek Instruments, Winooski, VT, USA).

#### 2.3.3. Individual Polyphenolic Compounds

Extracts of BB were analyzed with a Shimadzu Nexera I LC/MS-8045 (Kyoto, Japan) UHPLC system equipped with a quaternary pump and autosampler to which, respectively, an ESI probe and quadrupole rod mass spectrometer were applied. The separation was carried out on a Luna C18 reversed-phase column (150 mm × 4.6 mm × 3 mm, 100 Å), from Phenomenex (Torrance, CA, USA). During the analysis, the column was held at a temperature of 40 °C. A 100 L injection volume was utilized to determine the phenolic chemicals present in BB extracts. A gradient of ultrapure water and methanol was used as the mobile phase (Merck, Darmstadt, Germany). Formic acid was utilized as an organic modifier (Merck, Darmstadt, Germany). Initial gradient: methanol/water/formic acid water, 5:90:5. The formic acid and methanol were of LC/MS quality. The flow rate that was used was 0.5 mL/minute. A quadrupole rod mass spectrometer using electrospray ionization (ESI) was used for the detection in both the negative and positive MRM (multiple reaction monitoring) ion modes. The temperature at the interface was fixed at 300 °C. Gas nitrogen was utilized for vaporization and drying at 35 psi and 10 L/min, respectively. By comparing MS spectra and their transitions between the isolated compounds and standards, the identification was carried out. The major transition from the substance’s MS spectra served as the basis for identification and quantification. Calibration curves were used to conduct the quantification.

### 2.4. Green Synthesis of AgNPs Using Bee Bread Extracts

AgNPswere obtained by a one-step green synthesis, the BB extracts were mixed with 5 mM AgNO_3_ solution at a 1:5 ratio (*v*/*v*). The reaction was performed at room temperature and in natural light for 2 h. After 20–30 min, the color started to change from pale green-yellow until it reached a reddish-brown color. After 2 h, the reaction mixture was maintained in dark conditions for the next 24 h, centrifuged at 6500 rpm, washed three times with ultrapure H_2_O, and dried overnight at 40 °C in the oven to acquire the AgNPs in powder form. A similar synthesis method was published in [12] for bee pollen. AgNP samples of the desired concentration were obtained by dispersion in ultrapure water. All AgNP samples were sonicated for 10 min before characterization and assessment of biological effects.

### 2.5. Characterization of AgNPs

#### 2.5.1. UV–Vis Spectroscopy Analysis

The AgNPs formation was followed by recording the extinction spectra in the 300–700 nm range using a UV–Vis spectrophotometer (Synergy™ HT BioTek Instruments, Winooski, VT, USA). Additionally, the color change of the colloidal solution from pale green-yellow to reddish-brown is indicative of the metal ion reduction, thereby consequently confirming the successful synthesis of AgNPs.

#### 2.5.2. Dynamic Light Scattering and Zeta Potential

Nanoparticle stability was evaluated at room temperature by dynamic light scattering (DLS), while the surface zeta potential was measured by Nano ZS90 Zetasizer particle analyzer from Malvern Panalytical Ltd. (Worchestershire, UK), equipped with a He–Ne laser (633 nm, 5 mW) in a 90° configuration. All measurements were made in triplicate and average values were reported.

#### 2.5.3. TEM-EDX Analyses

Using a Hitachi HD2700 cold field emission gun STEM (Chiyoda, Tokyo, Japan) outfitted with two windowless EDX detectors (X-MaxN 100) from Oxford Instruments (Abingdon, UK) in high-vacuum mode (HV) and an acceleration voltage of 200 kV, the morphology of AgNPs was examined. To ensure accurate and representative measurements of the analyzed sample, copper was used as support for the AgNPs.

### 2.6. Determination of Antioxidant Activity

#### 2.6.1. Determination of DPPH Scavenging Activity (DPPH Method)

The scavenging activity of AgNPs against 2,2-diphenyl-1-picrylhydrazyl radical (DPPH) was evaluated spectrophotometrically by the method used by [51], with minor adjustments, and was adapted for use on a microplate reader. Briefly, 40 L of properly diluted extracts were combined with 200 L of DPPH solution (0.02 mg/mL). After 15 min, the absorbance of the samples were tested at 517 nm. A Trolox calibration curve was created for measurement with solutions ranging from 0.01 to 0.1 mM (R^2^ = 0.9988). The activity of radical scavenging is measured in milligram equivalent Trolox per gram of sample (mmol Trolox equivalent/g dry matter sample).

#### 2.6.2. Determination of Trolox Equivalent Antioxidant Capacity (TEAC Method)

Trolox equivalent antioxidant assay is based on the scavenging of the 2,2′-azinobis-(3-ethylbenzothiazoline- 6-sulphonic acid) radical (ABTS^•+^), converting it into a colorless product and was performed according to the method described by Cornea-Cipcigan et al. [52] with some modifications. The interaction between a 7 mM ABTS solution and a 2.45 mM potassium persulfate solution led to the formation of the ABTS^•+^ cation radical. The ABTS^•+^ solution was diluted with ethanol prior to measurement, resulting in an absorbance of 0.700 ± 0.025 at 734 nm. An amount of 30 mL of each sample and 170 µL of the resultant solution were combined for the test. After 6 min, the absorbance was measured.

The standard curve was linear between 0.04 and 0.4 mg Trolox (R^2^ = 0.9975). Results were expressed in milligrams of equivalent Trolox per gram of sample (mg Trolox equivalent/g dry matter sample).

#### 2.6.3. Determination of Ferric Reducing/Antioxidant Power (FRAP Method)

Ferric reducing/antioxidant power (FRAP) was performed by the method described by [53] with the adaptations imposed by the matrix studied. An amount of 10 µL of each BB extract was added to 300 µL of FRAP reagent and 10 µL of ultrapure water. The samples underwent a 5 min incubation at a temperature of 37 °C. The antioxidant capacity was calculated by comparing the reaction signal to known concentrations of aqueous Fe^II^ solutions (0.1–1 mmol/L of FeSO_4_·7H_2_O) and a standard calibration curve (R^2^ = 0.9966) was utilized. The absorbance was read at 593 nm and the results were expressed as mmol/g Fe^II^ dry matter sample.

### 2.7. Antimicrobial Activity Tests

The antimicrobial activity of the tested compounds was initially assessed by the disk diffusion method, followed the by microdilution method [54,55]. The selected strains were the following: *Staphylococcus aureus* (ATCC 25923), *Bacillus cereus* (ATCC 11778), *Enterococcus faecalis* (ATCC 29212), *Escherichia coli* (ATCC 25922), *Pseudomonas aeruginosa* (ATCC 27853), *Salmonella enteritidis* (ATCC 13076), and *Candida albicans* (ATCC 10231).

#### 2.7.1. Disk Diffusion Method

After preparing the microbial suspensions to a concentration of 0.5 McFarland, 0.5 mL of each suspension was inoculated on Petri dishes with MH agar plates and SDA agar for *Candida* species. Excess liquid was removed, and the agar surface was allowed to dry at 35 °C for 15–20 min. Afterward, wells were made aseptically, and exactly 20 µL of each sample was distributed into each well. As a positive control, an amoxicillin disk (30 μg/mL) was used for Gram-positive bacteria, a norfloxacin disk (10 μg/mL) for Gram-negative bacteria, and a miconazole disk (10 μg/mL) for yeast. Finally, plates were incubated at 37 °C for 24 h for bacteria and at 28 °C for 48 h for the fungal strain, and the diameters of the inhibition zones (in mm) were measured. The analysis was performed in triplicate.

#### 2.7.2. Determination of the Minimum Inhibitory Concentrations (MICs)

Minimum inhibitory concentrations (MICs) were determined by a serial microdilution method in Mueller–Hinton broth (supplemented according to species) using a final microbial suspension of 0.5 McFarland. Each sample was serially diluted in a range of 0.19 g/mL to 100 g/mL, with amoxicillin employed as a positive control for Gram-positive bacteria, norfloxacin for Gram-negative bacteria, and miconazole for yeast. Furthermore, untreated bacteria served as a negative control. The plate was examined at 600 nm with a BioTek Synergy 2 multichannel spectrophotometer (BioTek Instruments, Winooski, VT, USA) after 24 h of incubation at 37 °C. The MIC for each microorganism was considered the lowest concentration showing 100% of microorganism growth inhibition. The analysis was performed in triplicate.

#### 2.7.3. Bacterial Growth Assays

Bacterial growth assays were carried out using Urcan et al.’s [56] 96-well plate protocol with slight modifications, which allows the monitoring of the entire bacterial growth phase and aims to determine the lag phase length and slope during the logarithmic phase. A 100 µL volume of each sample of concentration MIC and 1/2 MIC were added to 100 µL broth medium (MHB) and were inoculated with 10 µL of the selected bacterial strains, whose optical density (OD600 nm) was previously adjusted to 0.001. The plates were incubated for 24 h at 37 °C in a BioTek Synergy 2 Multichannel Spectrophotometer (BioTek Instruments, Winooski, VT, USA). The optical density was taken every 15 min and bacterial growth curves were drawn. Positive control was represented by bacterial strains and MHB, the microbial cultures showed a growth curve according to the specific culture characteristics of the species. MHB was used as negative control. The growth of bacteria was compared to the positive and negative control curves. The test was performed in triplicate.

### 2.8. Cell Culture: MTT Assay

The experiments were performed on human tumor Caco-2 (colon adenocarcinoma, ATCC HTB-37) cell line. Caco-2 was grown in Eagle’s minimum essential medium (MEM) containing 2 mM L-glutamine, 1 mM sodium pyruvate, 1% (*v*/*v*) NEAA, 10% (*v*/*v*) fetal bovine serum (FBS), and 37 °C under an environment of 5% CO2 and 95% relative humidity. The cell line was detached at about 80% confluence using 0.25% (*w*/*v*) trypsin-0.53 mM EDTA solution and seeded in 96-well microplates at a concentration of 5 × 10^4^ cells per well in 200 µL culture medium. An amount of 1, 2, 4, 8, 16, 32, and 64 µg/mL of each BB-AgNPs sample were added to the culture medium after 24 h, and cells were incubated for the next 24 h under the same conditions. The impact of the cytotoxicity assay was evaluated using the 3-(4,5-dimethyl-2-thiazolyl)-2,5-diphenyl-2H-terazolium bromide reagent (MTT). Following the PBS washing step, cells were incubated for 1 h at 37 °C with a 150 µL/well MTT solution (5 mg/mL). The resultant formazan crystals were dissolved in dimethyl sulfoxide (DMSO) at a concentration of 150 µL/well. BioTek Synergy Instruments, Winooski, VT, USA, was used as microplate reader to detect absorbance at 550 and 630 nm. The percentage of the control (cells only incubated in normal media) was used to express the cell viability.

Concentrations of BB-AgNP samples showing a 50% reduction in cell viability (IC50 values) were then calculated. All experiments were conducted in triplicate.

### 2.9. Statistical Analysis

Statistical analyses were performed with the GraphPad Prism 9 statistics program. Data statistical analyses were achieved by using one-way ANOVA and Tukey’s test. The level of significance was set at *p* < 0.05.

## 3. Results

### 3.1. Characterization of BB Extracts Used for AgNPs’ Green Synthesis

Six aqueous extracts of BB that had different botanical origins, as shown in a previous study [32], were used to biosynthesize silver nanoparticles. UV–Vis spectrophotometric and LC/MS methods were used to identify if phenolic compounds may act as reducing agents in the biosynthesis of silver nanoparticles mediated by BB extracts (BB-AgNPs). Phenolic compounds represent a significant group of substances with numerous biological activities. In addition, to track whether these biomolecules are involved in the biosynthesis process and are responsible for the reduction in AgNPs, an analysis of the solution was conducted after the synthesis process was carried out. The total polyphenol (TPC) and flavonoid (TFC) contents of BB extracts and the remaining solution after the green synthesis of the BB-AgNPs were performed and are presented in Table 1.

The total polyphenolic content ranged between 7.95 ± 0.55 mg GAE/g and 21.46 ± 0.97 mg GAE/g in the BB aqueous extracts and a significant decrease was determined in the solutions that remained after the synthesis of the BB-AgNPs, where the values varied between 1.46 ± 0.09 and 2.69 ± 0.12 mg GAE/g. Additionally, in the case of total flavonoid content, the measured values were higher for BB extracts and they varied between 3.43 ± 0.32 and 14.32 ± 0.43 mg Qe/g, while the values of the solution that remained after the biosynthesis were included between 0.46 ± 0.17 and 2.03 ± 0.07 mg Qe/g. For both determinations, sample BB1 had the highest values.

Identification of phenolic compounds from BB aqueous extracts and from the remaining solution after the green synthesis of the AgNPs were made by LC/MS method based on the substance’s major transition in the MS spectrum. The obtained phenolic profiles consisted mainly of flavonoids, but phenolic acids were also present. The individual polyphenolic compounds are presented in Table 2 and Table 3 and represent the average of three determinations ± standard deviation.

In all analyzed BB samples, the flavonol kaempferol was identified inthe largest amount, and the values varied from 111.91 ± 0.24 (BB3) mg/mL to 309.63 ± 0.56 mg/mL (BB1). Other flavonoids such as quercetin, myrcetin, and luteolin were identified in high quantities in the analyzed samples. Among the phenolic acids, *trans*-p-coumaric acid and ellagic acid were identified as the largest amount. Sample BB1 presented the highest amount of polyphenolic compounds, but also the highest diversity, while sample BB4 was the lowest.

After the biosynthesis process, the measured value for different phenolic compounds such as ferulic acid, rosmarinic acid, luteolin-7-O-glucoside, or carnosol reached concentrations below the detection limit. Moreover, the values recorded for all other phenolic compounds were lower, which may indicate their reduction in the synthesis process. The highest values were also recorded in this case for flavonol kaempferol and varied between 59.11 ± 0.16 (BB5) and 157.58 ± 0.41 mg/mL (BB1), but the values were much lower compared to the values obtained for BB extracts. As in the case of TPC and TFC, all measured values for phenolic composition using LC/Ms were higher in BB aqueous extracts (Table 2) than for the remaining solutions after the green synthesis of the AgNPs (Table 3).

### 3.2. Synthesis of BB-AgNPs and Characterization

The silver nitrate solution (colorless) reacted with BB aqueous extracts and formed a light yellow-green color solution, which changed its color to a dark reddish-brown, thus proving the transformation of Ag+ silver ions in Ag^0^ and eventually the synthesis of AgNPs.

**Figure 1:**
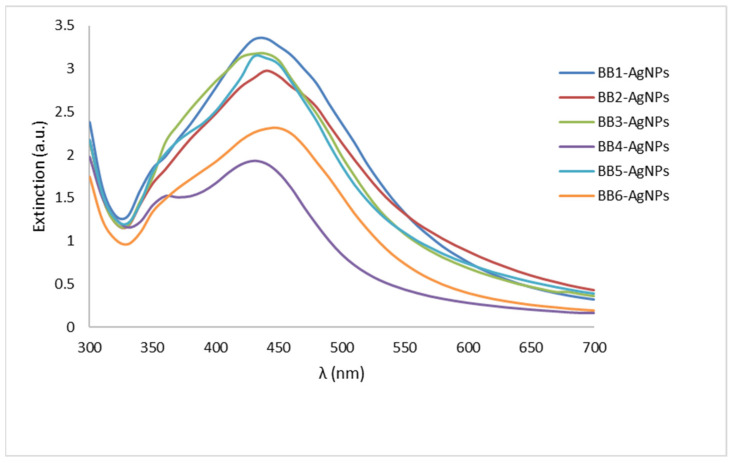
Extinction spectra of the as-biosynthesized BB-AgNPs.

Firstly, the synthesis of BB-AgNPs was confirmed by UV–Vis spectroscopy (Figure 1). On the spectra obtained by measuring the extinction in the range 300–700 nm, a plasmonic band between 400 and 450 nm was observed for each sample, which confirms the synthesis of nanoparticles. The intensity and position of the absorption peak can provide information about the size, shape, and concentration of the BB-AgNPs [57]. The results of the analysis showed a similar position of the plasmonic bands for all samples, but with different intensities.

The particle size and distribution of the biosynthesized BB-AgNPs were determined by dynamic light scattering (DLS) analysis (Table 4). DLS analysis showed that the nanoparticles obtained by green synthesis are polydisperse and sizes between 48.3 and 150.1 nm, which represent the average hydrodynamic diameter. The smallest size was obtained from the BB1-AgNPs sample at 48.3 nm, and the largest was from the BB4-AgNPs sample at 150.1 nm. The polydispersity index (PDI) describes the degree of uniformity and homogeneity of nanoparticles. The PDI values derived from DLS confirm the homogeneous distribution of the nanoparticles [12] and were included between 0.266 and 0.455. Further, zeta potential analyses were performed to determine the stability of the nanoparticles, and the values obtained were between +7.1 (BB5-AgNPs) and +13.3 mV (BB1-AgNPs) and are presented in Table 4.

**Figure 2:**
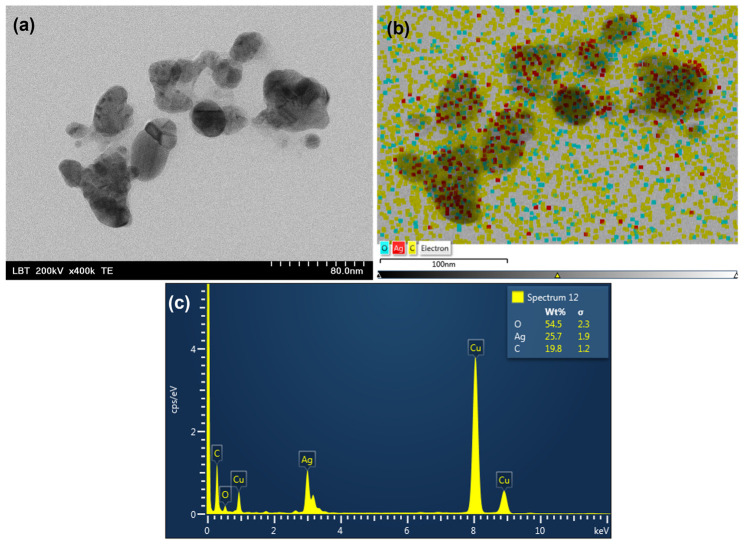
(**a**) Representative TEM analysis of the obtained BB1-AgNPs; (**b**) EDS layered image of BB1-AgNPs showing the distribution of the elements Ag (red points), O (yellow points), and O (blue points) in the formed nanoparticles; (**c**) EDX profile of bio-synthesized BB6-AgNPs.

**Figure 3:**
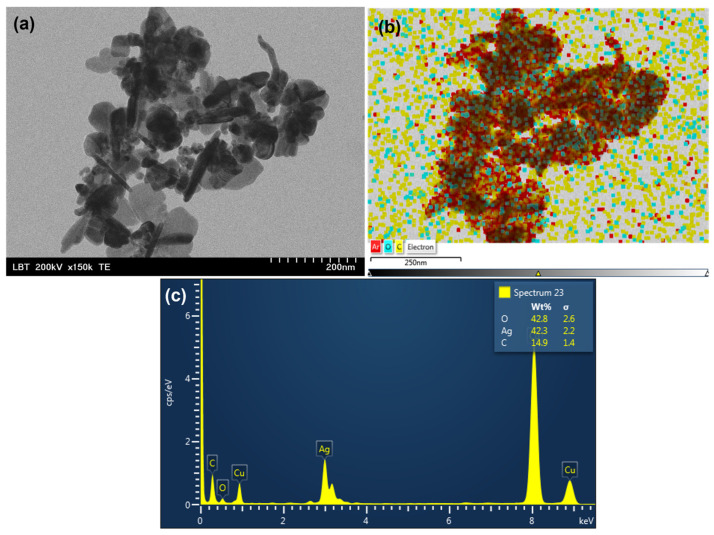
(**a**) Representative TEM analysis of the obtained BB6-AgNPs; (**b**) EDS layered image of BB6-AgNPs showing the distribution of the elements Ag (red points), O (bluepoints), and O (yellow points) in the formed nanoparticles; (**c**) EDX profile of biosynthesized BB6-AgNPs.

Transmission electron microscopy (TEM) was carried out to determine the morphology of nanoparticles formed by green synthesis mediated by BB extracts and to confirm the size. Figure 2a and Figure 3a show representative TEM images of samples BB1-AgNPs and BB6-AgNPs obtained by green synthesis, while Figure 2b,c and Figure 3b,c present the atomic composition of BB1-AgNPs and BB6-AgNPs. The micrographs obtained showed that the obtained silver nanoparticles have an irregular quasi-spherical and elongated shape.

For identifying the composition of biosynthesized nanomaterials, EDX analysis was performed. The result clearly shows that the biosynthesis of nanomaterials using BB extracts was successful. The EDX profile (Figure 2b,c and Figure 3b,c) recorded for the BB-AgNP samples shows the existing atoms in the sample such as carbon (C), oxygen (O), and silver (Ag) identified by the peak amplitude. It can be seen that the silver signal is stronger than the carbon signal. The signals for O and C are residues originating from the BB extract. The copper (Cu) signal is due to the sputter-coated sample for better conductivity. Additionally, Figure 2b and Figure 3b, which show the distribution of the elements in the nanoparticles formed, confirms the presence of AgNPs.

### 3.3. Antioxidant Activity of BB-AgNPs

The antioxidant activity of the green synthesized AgNPs obtained from the BB aqueous extracts was assessed by DPPH, ABTS, and FRAP assays. The obtained results are presented in Table 5.

Within the DPPH method, the values obtained varied between 6.71 ± 1.04 and 20.54 ± 0.46 mg Trolox equivalent/g with the best inhibition effect of the free radical 2,2-diphenyl-1-picrylhydrazyl determined at sample BB1-AgNPs. In the case of the ABTS method, the values obtained varied between 29.81 ± 1.63 and 56.82 ± 0.72 mg Trolox equivalent/g, and the BB1-AgNPs sample had the best effect. Ferric reducing/antioxidant power recorded values between 3.97 ± 0.62 and 12.17 ± 1.23 mmol/g Fe^II^, and sample BB1-AgNPs had the best results in this case as well. The differences obtained between the antioxidant activity values within the same method are most likely due to the different types of bioactive compounds involved in biosynthesis. All tested BB-AgNPs showed good antioxidant activity, but among the three methods tested, they had the highest efficiency against ABTS free radicals.

### 3.4. Evaluation of Antimicrobial Activity of BB-AgNPs by Disk Diffusion Method

The disk diffusion method was used as a preliminary method to identify the antimicrobial activity of AgNPs against different Gram-positive and Gram-negative bacterial strains: *Staphylococcus aureus* (ATCC 25923), *Bacillus cereus* (ATCC 11778), *Enterococcus faecalis* (ATCC 29212), *Escherichia coli* (ATCC 25922), *Pseudomonas aeruginosa* (ATCC 27853), *Salmonella enteritidis* (ATCC 13076), and on yeast, *Candida albicans* (ATCC 10231). Following this test, a strong antimicrobial effect was observed on all tested microorganisms and the inhibition diameters are detailed below, in Table 6.

The results showed good inhibitory effects on both Gram-positive and Gram-negative bacteria and yeast. Among the Gram-positive *E. faecalis* was the most sensitive to the action of the tested AgNPs, and from the Gram-negative bacteria *P. aeruginosa*. Regarding the samples of BB-AgNPs, given the Gram-positive bacteria, the largest inhibition diameters were observed for the BB6-AgNPs, and from the Gram-negative bacteria, the largest inhibition diameters were observed for the BB1-AgNPs sample. In the case of the *C. albicans* strain, no significant differences were observed between the inhibition diameters of the BB1-AgNPs and BB6-AgNP samples.

It is important to note that the disk diffusion method is qualitative and may not provide exact quantitative results. The method also has some limitations, such as the potential for variability in the diffusion rate of the AgNPs in the agar, and the susceptibility of the microorganisms to the AgNPs. Therefore, it is recommended to complement the disk diffusion method with other methods, such as MIC determination, to obtain a more comprehensive assessment of the antimicrobial activity of BB-AgNPs.

### 3.5. Evaluation of Antimicrobial Activity of BB-AgNPs by MIC

The minimum inhibitory concentration (MIC) of silver nanoparticles (AgNPs) refers to the lowest concentration of the nanoparticles that is required to inhibit the growth of a specific microorganism. MIC values are commonly used to evaluate the antimicrobial activity of AgNPs and to determine their potential as alternative antimicrobial agents to traditional antibiotics. The MIC of each sample was achieved by the serial microdilution method and the results are presented in Table 7.

In the case of all samples, small concentrations were needed to observe the inhibitory effect, the obtained results showed that MIC values varied between 0.39 and 6.25 µg/mL. In terms of both Gram-positive and Gram-negative bacteria, the sample BB1-AgNPs, which had the lowest MIC value, showed the best results. For *C. albicans*, samples BB1-AgNPs and BB1-AgNPs presented the lowest MIC values at 0.39 µg/mL.

**Figure 4:**
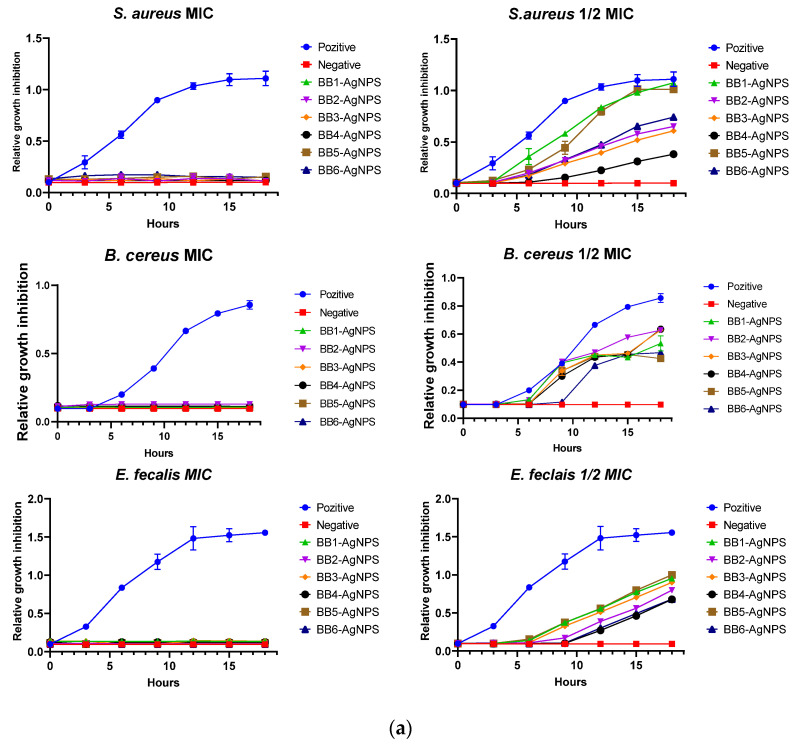

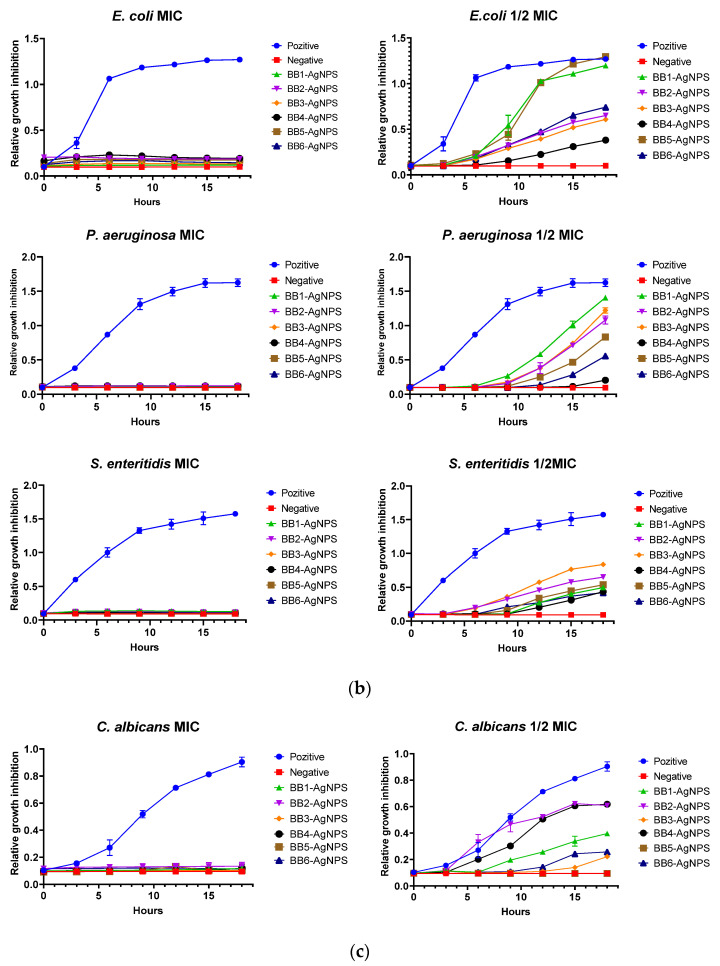
(**a**) Relative growth inhibition (means ± SD) of ½ MIC and MIC of BB-AgNPs on the growth of Gram-positive bacteria—*S. aureus*, *B. cereus*, *E. faecalis*—compared to the natural growth curve of each bacterial strain (blue). (**b**) Relative growth inhibition (means ± SD) of ½ MIC and MIC of BB-AgNPs on the growth of Gram-negative bacteria—*E. coli*, *P. aeruginosa*, *S. enteritidis*—compared to the natural growth curve of each bacterial strain (blue). (**c**) Relative growth inhibition (means ± SD) of ½ MIC and MIC of BB-AgNPs on the growth of *C. albicans* compared to the natural growth curve of each bacterial strain (blue).

A time-killing assay was carried out to better understand the BB-AgNPs’ kinetic effect against the examined microorganisms. The effect of BB-AgNPs at different concentrations on bacterial growth is presented in Figure 4a–c.

For all studied bacteria, the findings indicated exponential bacterial growth over time without treatment (positive) and a suppression of bacterial growth at MIC concentration. A ½ × MIC value of BB-AgNPs decelerated the growth of all tested microorganisms by decreasing the slope and extending the lag phase of the growth in a concentration-dependent manner. From Figure 4a–c, it can be observed how, depending on the strain and the sample, the time of the deceleration of bacterial growth is different. This, as well as the different microbial effects between the samples, are due to the different types of bioactive compounds involved in biosynthesis, which led to differences in AgNPs obtained in each sample, the different contact surfaces, as well as the different sizes.

### 3.6. Antiproliferative MTT Assay

MTT assay is a commonly used cell viability assay that measures the ability of cells to convert MTT (3-(4,5-dimethylthiazol-2-yl)-2,5-diphenyl tetrazolium bromide) to formazan, a colored product that reflects the metabolic activity and viability of cells. The cytotoxic effect of the BB-AgNPs was assessed by an MTT test, and the results were described as a percentage of the control (untreated cells).

**Figure 5:**
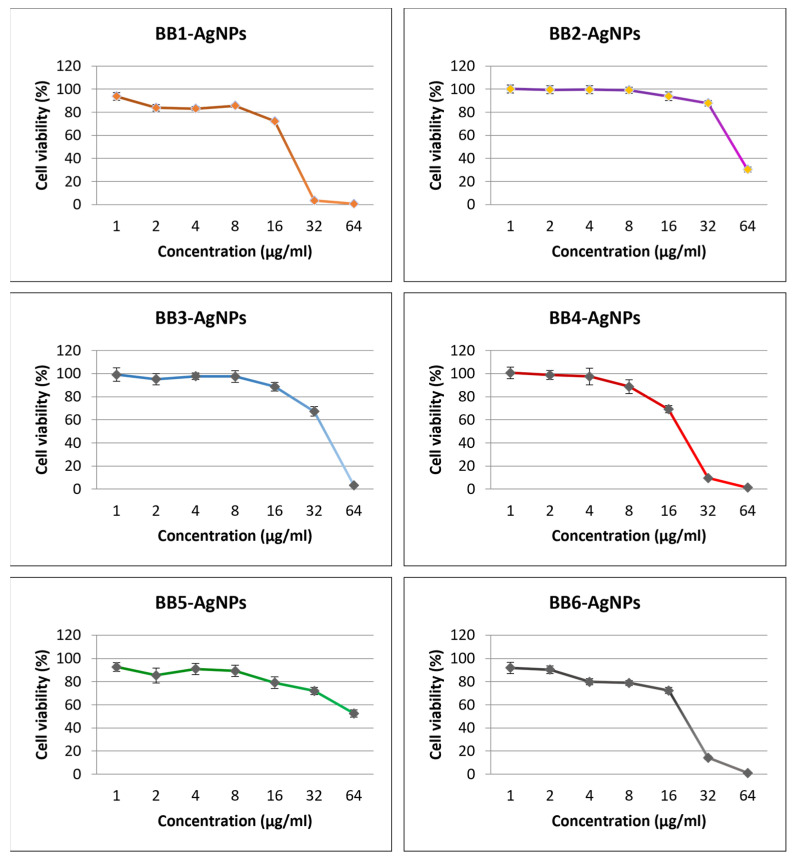
Cell viability after 24 h of treatment with the six samples of BB-AgNPs (1, 2, 3, 8, 16, 32, 64 µg/mL). The results are expressed as cell viability percentage (%).

Figure 5 shows the results of cytotoxicity tests performed on the Caco-2 cell line. Three out of six BB-AgNP samples showed a similar cytotoxic pattern (BB1-AgNPs, BB4-AgNPs, and BB6-AgNPs). The highest cytotoxic effect was observed in the sample BB1-AgNPs with a corresponding IC50 value equal to 24.58 µg/mL, while the lowest cytotoxic effect was observed in the case of BB5-AgNPs with an IC50 value of 67.91 µg/mL. The other IC50 values obtained, in descending order of cytotoxicity, were: 25.42 µg/mL for BB6-AgNPs, 27.54 µg/mL for BB4-AgNP, 37.22 µg/mL for BB3-AgNPs, and 53.39 µg/mL for BB2-AgNPs.

Group comparison was performed using one-way analysis of variance (ANOVA) followed by Tukey’s test. A *p*-value of ≤0.05 was considered to be of statistical significance (* *p* ≤ 0.05; ** *p* ≤ 0.01; **** *p* ≤ 0.0001). The most significant differences were observed in the concentration of 32 µg/mL for AgNPs-BB.

## 4. Discussion

Finding new potential sources to battle diverse infections is required nowadays owing to the emergence of multidrug-resistant bacteria or the harmful effects connected with the use of current medications. Nanotechnology plays a crucial part in modern medicine, offering several benefits for the treatment of human illnesses via target-oriented methods [35]. Nanoparticles, and especially AgNPs, due to their exceptional biocompatibility and long-term stability, are considered to be a good alternative to antibiotics, with a high potential to fight against multidrug-resistant bacteria [5,17,58,59].

In this study, green synthesis was used to produce AgNPs using six aqueous extracts of BB as reducing agents. In contrast to chemical and physical processes, the green synthesis of AgNPs uses plant-based compounds such as polyphenols, flavonoids, terpenes, and alkaloids as capping, reducing, and stabilizing agents to regulate the size and prevent the formation of agglomerated biocompatible nanoparticles. The existing studies in the literature show that different plant extracts, microorganisms, or bee pollen were used for the green synthesis of silver nanoparticles [4,8,9,10,11]. Therefore, to confirm the presence of bioactive compounds, which, according to the literature [60,61,62], act as reducing agents, the TPC and TFC of the BB extracts were measured. LC/MS analysis was also performed to identify the existing individual polyphenolic compounds. In addition, to track whether these biomolecules are involved in the biosynthesis process and are responsible for the reduction in AgNPs, an analysis of the solution was performed after the synthesis process was carried out.

The results obtained for the BB extracts showed values between 7.95 ± 0.55 and 21.46 ± 0.97 mg GAE/g (dry weight) for TPC, while for TFC, the values varied in the range between 3.43 ± 0.32 and 14.32 ± 0.43 mg Qe/g (dry weight). These variations are due to the different botanical origins of the BB samples, as has been shown in other studies [49,63].

Regarding the individual phenolic compounds identified in BB samples, the data obtained revealed a complex heterogeneous mixture of water-extracted metabolites, among which, kaempferol was found in the largest amount in all samples, whereas flavones such as apigenin, chrysene, luteolin, quercetin, rutin, and naringenin were found in smaller quantities. Likewise, phenolic acids such as caffeic, chlorogenic, fumaric, ellagic, salicylic acid, and diterpene carnosol were identified in the BB samples in variable amounts, as can be seen in Table 2. These phenolic compounds are normally found in BB and other bee products in variable amounts depending on the botanical or geographical origin and make these products possible sources of bioactive substances as previously other authors have shown [33,34,64,65]. Phenolic compounds represent a real fingerprint of bee products [49], through which the botanical origin can be identified.

After the AgNps synthesis, the remaining solution was analyzed in the same way, the values for TPC varied between 1.46 ± 0.09 and 2.48 ± 0.14 mg GAE/g (dry weight), and for TFC, the values ranged between 0.46 ± 0.17 and 2.03 ± 0.07 mg Qe/g (dry weight). The results showed a significant decrease in TPC and TFC compared to the values obtained for BB extracts, which indicates the reduction in these compounds during synthesis. These types of differences are also supported by other authors [66]. Large differences were also observed in the case of individual phenolic composition where all compounds presented a lower value or had values below the detection limit, such as ferulic and rosmarinic acid, luteolin-7-o-glycoside, naringenin, or carnosol. The greatest reduction in the quantity was observed in the case of kaempferol, which was initially the most abundant in all BB samples. In the BB1 sample, where kaempferol was also found in the largest quantity, the amount decreased from 309.63 ± 0.56 mg/mL to 157.58 ± 0.41 mg/mL.

In the case of this study, the exact mechanism of AgNPs formation is not known, but given the results mentioned above, where different polyphenols, flavonoids, and flavonols were identified, it can be suggested that these substances could be responsible for the synthesis of nanoparticles [67]. The reduction in the quantity of polyphenols and flavonoids, presented in Table 1, Table 2 and Table 3, after the synthesis process could support this theory. These results may indicate which were the main phenolic compounds involved in the reduction in silver ions. One flavonol involved in the reduction in silver ions process could be kaempferol, given the fact that a high reduction in its quantity was observed, but further studies are required. Additionally, according to some authors [44], the afore mentioned components can form repulsive forces among the silver nanoparticles, which are responsible for the stabilization and prevention of aggregation in the solution of nanoparticles.

Each sample of AgNPs exhibits a characteristic broad and strong single-surface plasmon resonance band of 400–450 nm (Figure 1), as follows: BB1-AgNPs: 436 nm; BB2-AgNPs: 444 nm; BB3-AgNPs: 438 nm; BB4-AgNPs: 439 nm; BB5-AgNPs: 445 nm; and BB6-AgNPs: 440 nm. These results are in agreement with those obtained by other authors for AgNPs obtained by green synthesis using pollen or plant extracts as mediators [51,66]. The results of the analysis showed a similar position of the plasmonic bands for all six BB-AgNP samples, located in the visible range of the electromagnetic spectrum with a maximum absorbance at around 400–450 nm, but with different intensities.

The values obtained for the size of nanoparticles by DLS analysis were between 48.3 nm (BB1-AgNPs) and 150.1 nm (BB4-AgNPs); these values are higher than those described in the literature for nanoparticles synthesized with pollen extract [12,13]. These size differences may be due to the different types of reducing agents present in the BB extracts used. It was observed that sample BB1, which had the highest amount of kaempferol, resulted in the formation of BB1-AgNPs, which had the smallest size at 48.3 nm. In addition, sample BB1-AgNPs had the best antioxidant and antimicrobial effect. In the case of the other samples, no correlation was observed between the total amount of phenolic compounds and the particle size, but in general, the samples that had lower concentrations of phenolic compounds led to the formation of AgNPs with dimensions greater than 100 nm. The potential stability and behavior of BB-AgNPs in solution depend on the value of zeta potential; in our study, zeta potential values were between +7.1 and +13.9 mV, so the particles were considered stable, but over time, they tend to form aggregates.

TEM analysis showed nanoparticles of irregular, quasi-spherical, and elongated shapes for all analyzed samples. The results observed in the TEM analysis are largely correlated with those from DLS, but it was observed that the values obtained in the DLS analysis are slightly higher compared to those observed in the TEM analysis. DLS measurements may include the hydrodynamic size of AgNPs and any agglomerates or aggregates present in the solution, while TEM images provide a more accurate evaluation of the specific shape and size of individual nanoparticles [68,69]. Similar forms were obtained by other authors for the synthesis mediated by bee pollen [12]. Studies have shown that spherical AgNPs have high antimicrobial activity due to their high surface area-to-volume ratio [70]. The small size of spherical AgNPs allows them to easily penetrate microbial cell walls, causing damage to the cell membrane and eventually leading to cell death [71].

The EDX profile revealed that the compounds identified in BB-AgNP samples were C, Ag, O, and Cu and the EDS layered images pointed out the distribution of these elements in the nanoparticles. C and O originate from the BB extracts and Cu is present due to the sputter coating of the sample. Our results concur with those mentioned in the literature [1,12].

Antioxidant activity of the obtained AgNPs by mediation with the BB extracts against free radicals 2,2-diphenyl-1-picrylhydrazyl radical (DPPH), 2,2′-azinobis-(3-ethylbenzothiazoline- 6-sulphonic acid) radical (ABTS^•+^) and total antioxidant potential by ferric reducing ability (FRAP) were studied. Sample BB1-AgNPs had the best effect with a value of 56.64 ± 0.35 mg Trolox equivalent/g against the ABTS^•+^ radical, followed by sample BB6-AgNPs with 42.82 ± 0.72 mg Trolox equivalent/g. The lowest effect was recorded for the sample BB4-AgNPs, with a value of 29.81 ± 1.63 mg Trolox equivalent/g. The values obtained against the DPPH radical were lower compared to those against the ABTS^•+^ radical, but sample BB1-AgNPs still had the best effect and showed an antioxidant value of 20.54 ± 0.73 mg Trolox equivalent/g, while sample BB4-AgNPs had the lowest inhibitory effect. The values for total antioxidant potential were the lowest, and again, BB1-AgNPs presented the best ferric-reducing ability, and it was 12.34 ± 0.33 mmol/g Fe^II^. Overall, the best inhibitory effect was observed against ABTS^•+^ free radical. Similar results were observed by Turunc et al. [12] for AgNPs obtained using bee pollen and by Sreelekha et al. [70] for silver nanoparticles obtained using leaf extract of *M. frondosa*.

The increased antioxidant activity of biosynthesized AgNPs can be attributed to the presence of phytochemical residues in the pollen content of BB acting as a capping agent [71]. This is also confirmed by Sreelekha et al. [70] who reported that AgNPs obtained by green synthesis displayed superior antioxidant activity compared to the chemically synthesized one because of the presence of a bioactive capping agent on its surface. The differences between the antioxidant activity values by different methods are due to the different mechanisms of action and the different solubility of each free radical. DPPH assay evaluates the ability of the samples to donate hydrogen to the free radical and is suitable for hydrophilic systems, while the ABTS^•+^ assay is an electron transfer method and can react both in hydrophilic and hydrophobic systems, which will result in greater values [72].

The antimicrobial activity of BB-AgNPs was evaluated against both Gram-positive and Gram-negative bacterial strains and yeast strains using different microbiological assays. In the case of the disk diffusion method, all six BB-AgNP samples showed high antimicrobial activity against the tested bacteria, showing inhibition zones in a range of 7.67 ± 0.59 and 22.21 ± 1.06 mm. BB6-AgNPs presented the highest inhibition diameters for Gram-positive bacteria, while BB1-AgNPs for Gram-negative bacteria. The best results were observed on Gram-negative strains, and *E. coli* and *P. aeruginosa* were the most sensitive, while among the Gram-positive ones, *E. faecalis* presented the largest diameters of inhibition. The fact that Gram-negative bacteria were more sensitive to AgNPs is also confirmed by other authors [73] due to the differences between the cell walls. These results, together with the data obtained by MIC assay, indicate that AgNPs had a strong antimicrobial action even at very low concentrations. The MIC values obtained varied between 0.39 and 6.25 µg/mL depending on the type of microorganism and the BB-AgNPs sample. For *P. aeruginosa*, a strain that was the most sensitive in this study, MIC values ranged between 0.39 and 1.56 µg/mL. Similar values were obtained by Yu-Guo Yuan et el. [74], who tested the antimicrobial effect of AgNPs synthesized by quercetin mediation. It was observed that sample BB1-AgNPs, which had the smallest size, had the lowest MIC values, which were between 0.39 and 0.78 µg/mL, but also the largest amount of kaempferol in the BB1 extract. Therefore, the bioactive compounds present on the surface of the synthesized green nanoparticles may be responsible for the enhanced antimicrobial activity of the AgNPs. Similar results were obtained by Al-Yousef et al. [13], who analyzed AgNPs obtained by mediation with bee pollen and reported MIC values between 1.75 and 6.75 µg/mL. Most existing studies have analyzed the antimicrobial activity of aqueous or alcoholic BB extracts [33,40,75] against different types of microorganisms, but the results presented indicate a much lower antimicrobial activity than that of BB-AgNPs. AgNPs due to their small size and large contact surface, which allows for a greater interaction with bacterial cell membranes and a more effective release of silver ions, generally exhibit a higher antibacterial activity compared to larger particles [76]. The shape of AgNPs has also been found to influence their antibacterial activity. For example, AgNPs with a spherical shape have been found to exhibit a higher antibacterial activity compared to rod-shaped or triangular particles [77]. BB1-AgNPs sample that showed the best antibacterial effect on Gram-negative bacteria also had the smallest size and had a quasi-spherical shape.

According to the literature, AgNPs obtained by green synthesis have antimicrobial activity against a wide variety of microorganisms, including Gram-negative and Gram-positive bacteria, such as *S. aureus*, *E. coli*, *P. aerugionosa*, *E. faecalis* [78,79,80], *Staphylococcus epidermidis* [81], *S. pyogenes* [82], *K. pneumoniae*, *S. typhi*, *P. vulgaris* [17,83], *S. typhimurium* [84], and *B. cereus*, *B. subtilis* [85], and the minimum inhibitory concentrations varied between 0.25 and 12 µg/mL. The results obtained in this study were consistent with the literature data, despite differences found due to the bacterial strains investigated or AgNPs’ type, size, or shape.

For *C. albicans* yeast, MIC values were between 0.39 and 3.12 µg/mL. Furthermore, BB1-AgNPs and BB5-AgNPs presented the lowest MIC values, which indicates a good antifungal activity of BB-AgNPs. Similar results were also observed in other studies [81].

The MIC values of AgNPs vary depending on several factors, such as the size, shape, surface chemistry, and concentration of the nanoparticles, as well as the type and strain of the microorganism being tested. In general, lower MIC values indicate a greater antimicrobial activity and efficacy of AgNPs [18]. The exact action mechanism of AgNPs against microorganisms is not yet fully elucidated. It is a debate whether AgNPs act directly on the cell wall of the microorganisms or only induce a cytotoxic effect [77]. Bondarenko et al. established a synergistic antibacterial impact between two antibacterial pathways [86]. The existing data in the literature show a broad antibacterial spectrum of AgNPs, including bacterial strains with antibiotic resistance [68,69,70,71]. Several studies have reported the effectiveness of AgNPs against antibiotic-resistant bacteria, such as methicillin-resistant *S. aureus* (MRSA), vancomycin-resistant *Enterococcus* spp., and carbapenem-resistant *Acinetobacter baumannii* (CRAB) [87]. A study published by da Cunha et al. [88] reported that AgNPs were effective against MRSA strains and could reduce the expression of virulence factors associated with bacterial pathogenesis. Another study reported that AgNPs were effective against CRAB and could inhibit biofilm formation, a major contributor to antibiotic resistance [69]. AgNPs have also been shown to enhance the activity of conventional antibiotics, making them more effective against antibiotic-resistant bacteria such as *E. coli* [89]. Despite the promising antimicrobial activity of AgNPs, the development and application of these nanoparticles as antimicrobial agents still face some challenges, such as the potential for toxicity to plants, animals, and human cells; for this reason, a comprehensive toxicological evaluation is necessary [18].

Several studies have reported the use of the MTT assay to evaluate the cytotoxicity of AgNPs in vitro, with varying results depending on the size, shape, surface chemistry, and concentration of the nanoparticles, as well as the type and origin of the cells being tested [90]. Generally, a lower cell viability and a higher cytotoxicity are observed with increasing concentrations and exposure times of AgNPs [77,91]. The IC50 values for the biosynthesis of AgNPs were between 25 µg/mL and 70 µg/mL in the study by Han et al. [92] on human lung epithelial adenocarcinoma cell line, while Kis et al. [1] revealed IC50 values of AgNPs obtained by green synthesis with *Populi gemmae* extract between 4.39 µg/mL and 16.14 µg/mL for lung adenocarcinoma cell line A549. For breast cancer cells, the same authors obtained IC50 values between 40.23 µg/mL and 48.92 µg/mL.

Regarding the effect of AgNPs on non-cancerous cells, the existing studies in the literature [91,93] show that AgNPs can exhibit dose-dependent toxicity, generate an inflammatory response, and induce cellular oxidative stress and mitochondrial dysfunction. Cell viability can also be influenced by factors such as the type of treated cells, the time of exposure, size, and shape of AgNPs.

In this study, BB-AgNPs toxicity was tested on the human colon adenocarcinoma cell line using an MTT assay. The obtained results showed that samples BB1-AgNPs, BB4-AgNPs, and BB6-AgNPs had a similar cytotoxic pattern, but sample BB1-AgNPs, which had the smallest size at 48.3 nm and a quasi-spherical shape, presented the strongest cytotoxic effect, with a corresponding IC50 value equal to 24.58 µg/mL. Studies have shown that the size and shape of AgNPs can significantly impact their anticancer activity [1]. Spherical silver nanoparticles (AgNPs) have been shown to have cytotoxic effects on various types of cells, including normal and cancerous cells. Moreover, the cytotoxicity of AgNPs is also dependent on their concentration and exposure time. Higher concentrations of AgNPs and longer exposure times have been found to increase their cytotoxicity toward cells. The lowest cytotoxic effect was observed in the case of BB5-AgNPs and their IC50 had a value of 67.91 µg/mL. In addition, BB1-AgNPs had the highest positive charge among all samples. Liao and colleagues [77] mentioned that the surface charge of AgNPs can also affect their cytotoxicity, and that positively charged nanoparticles are more cytotoxic than negatively charged or neutral ones. This is because positively charged nanoparticles can interact more strongly with negatively charged cellular components such as cell membranes and intracellular proteins.

The results obtained were in line with those reported by other authors for the human tumor Caco-2 colon adenocarcinoma cell line [94,95,96]. A study conducted by Gomaa [15] on the colon carcinoma cell line showed toxicity at much lower concentrations, the IC50 value of the tested AgNPs had a range between 2.3 and 2.2 µg/mL. Buttacavoli et al. also investigated the cytotoxic effect of AgNPs bio-generated using *Klebsiella oxytoca* on three human colon cancer cell lines HT-29 and HCT 116 [97], and the results obtained showed IC50 values of 20 ± 2 µg/mL, 26 ± 2 µg/mL, and 34 ± 4 µg/mL, respectively. These values are comparable to those obtained for the samples analyzed in this study and were found to be within the clinically acceptable concentration of 100 μg/mL [98], suggesting a potential anticancer effect of AgNPs. It is important to understand the mechanisms of AgNPs that lead to the anticancer effect; according to the literature, AgNPs can induce apoptosis in colon cancer cells through various mechanisms, including oxidative stress, inhibit angiogenesis, and prevent cell division by DNA damage [99]. Furthermore, AgNPs have been documented to modulate the expression of proteins and key enzymes and are involved in glucose metabolism, including glycolysis and the tricarboxylic acid cycle. These changes in metabolic pathways can disrupt the energy production and nutrient utilization of colon cancer cells, leading to growth inhibition and apoptosis. Recently, there is a great deal of emphasis on identifying potential biomarkers in the diagnosis and treatment of cancer [100]. The application of colon cancer metabolism study holds great potential for providing further significance to the observed antiproliferative effects of the obtained AgNPs against colon cancer. Investigating the metabolic alterations in colon cancer cells in response to AgNPs can shed light on the underlying mechanisms of their antiproliferative activity and identify specific metabolic pathways that are affected [101].

To the best of our knowledge, no prior studies have investigated the in vitro antioxidant, antimicrobial, and antiproliferative effect on the human colon adenocarcinoma cell line of the green synthesized silver nanoparticles obtained from BB aqueous extracts. As a consequence, the acquired results appear promising because the usage of AgNPs is regarded an efficient antibacterial and anticancer approach. Nevertheless, further research of these early data is necessary to understand the specific mechanism of action.

## 5. Conclusions

The biosynthesis of AgNPs was achieved successfully using BB aqueous extract as a reducer. The physicochemical investigation revealed that the BB-AgNPs were polydisperse, stable, and small, making them appropriate for biological applications. The obtained results showed good antioxidant activity of AgNPs against DPPH, ABTS, and FRAP free radicals. All BB-AgNPs exhibited potent antimicrobial activity against Gram-positive bacteria *S. aureus B. cereus*, *E. faecalis*, Gram-negative bacteria *E. coli*, *P. aeruginosa*, *S. enteritidis*, and yeast *C. albicans*. Tested BB-AgNP samples elicited a dose-dependent antiproliferative potential on human colon adenocarcinoma cell line. Sample BB1-AgNPs was synthesized from the BB extract with the highest amount of polyphenolic compounds, especially kaempferol, leading to the obtaining of quasi-spherical nanoparticles with an average size of 48.3 nm and a zeta potential of +13.9 mV. Altogether, these led to the best antioxidant and antimicrobial effect against Gram-negative bacteria, but also to the highest cytotoxicity. In the case of the other samples of BB-AgNPs, no correlation could be established between the aspects mentioned above. Further studies are required to develop suitable technology based on BB extracts to obtain nanoparticles with specific sizes and morphology to be used in pharmaceutical and biotechnological applications. The in vitro results obtained following this investigation can provide important insights into the potential therapeutic effects of the tested AgNPs on colon adenocarcinoma cells and may lay the foundation for further research and potential translation into clinical applications, providing promising perspectives for developing targeted therapies and improving patient outcomes in the treatment of colon cancer.

## Figures and Tables

**Table 1:** The total phenolics and flavonoid content of aqueous BB extracts and the remaining solution after the green synthesis of the BB-AgNPs.

	Sample	BB Extracts (A)	Solution Remained after the Biosynthesis of AgNPs (B)	A:B
TPC (mg GAE/g) dry weight	BB1	21.46 ± 0.97 ^a^	2.48 ± 0.14 ^b^	8.65
	BB2	11.65 ± 0.41 ^a^	1.27 ± 0.16 ^b^	9.17
	BB3	13.71 ± 0.63 ^a^	2.69 ± 0.12 ^b^	5.09
	BB4	14.33 ± 0.35 ^a^	1.74 ± 0.08 ^b^	8.23
	BB5	7.95 ± 0.55 ^a^	1.46 ± 0.09 ^b^	5.46
	BB6	15.48 ± 0.24 ^a^	2.31 ± 0.18 ^b^	6.70
TFC (mg Qe/g) dry weight	BB1	14.32 ± 0.43 ^a^	2.03 ± 0.07 ^b^	7.06
	BB2	4.74 ± 0.03 ^a^	1.43 ± 0.15 ^b^	3.31
	BB3	9.02 ± 0.29 ^a^	1.96 ± 0.11 ^b^	4.60
	BB4	3.43 ± 0.32 ^a^	0.46 ± 0.17 ^b^	7.45
	BB5	9.13 ± 0.14 ^a^	1.71 ± 0.09 ^b^	5.33
	BB6	5.22 ± 0.19 ^a^	0.69 ± 0.05 ^b^	7.56

GAE—Gallic acid equivalents; QE—quercetin equivalents; values are shown as mean ± standard deviation. Within the same row, different letters indicate significant differences (*p* < 0.05).

**Table 2:** Polyphenolic composition of BB aqueous extracts.

Identified Compound, mg/mL (dw)	Sample					
	BB1	BB2	BB3	BB4	BB5	BB6
Caffeic acid	1.66 ± 0.05 ^b^	2.09 ± 0.11 ^a^	2.07 ± 0.06 ^a^	0.49 ± 0.04 ^c^	nd	2.73 ± 0.08 ^a^
Chlorogenic acid	0.20 ± 0.07 ^c^	4.73 ± 0.14 ^a^	3.14 ± 0.08 ^b^	0.13 ± 0.02 ^c^	3.58 ± 0.09 ^b^	0.18 ± 0.02 ^c^
*trans*-p-coumaric acid	13.70 ± 0.04 ^b^	46.04 ± 0.21 ^c^	20.81 ± 0.11 ^d^	10.06 ± 0.17 ^e^	0.17 ± 0.02^f^	56.03 ± 0.11 ^a^
Ellagic acid	21.91 ± 0.11 ^c^	nd	nd	49.37 ± 0.20 ^d^	156.47 ± 0.36 ^a^	137.08 ± 0.28 ^b^
Ferulic acid	nd	6.34 ± 0.10 ^b^	7.84 ± 0.15 ^a^	nd	nd	nd
Rosmarinic acid	nd	0.06 ± 0.01 ^a^	0.07 ± 0.02 ^a^	nd	0.18 ± 0.04 ^a^	0.18 ± 0.02 ^a^
Salicylic acid	2.71 ± 0.05 ^e^	20.04 ± 0.15 ^a^	9.23 ± 0.10 ^b^	17.53 ± 0.12 ^c^	1.49 ± 0.07 ^d^	3.09 ± 0.13 ^e^
Apigenin	0.44 ± 0.09 ^b^	1.76 ± 0.08 ^a^	1.06 ± 0.08 ^c^	0.67 ± 0.08 ^d^	0.61 ± 0.04 ^d^	1.86 ± 0.12 ^a^
Chrysine	0.63 ± 0.05 ^b^	0.54 ± 0.08 ^b^	0.31 ± 0.02 ^c^	0.33 ± 0.02 ^c^	0.95 ± 0.07 ^a^	0.69 ± 0.08 ^b^
Hyperoside	1.24 ± 0.07 ^e^	0.65 ± 0.02 ^b^	1.63 ± 0.08 ^a^	0.59 ± 0.08 ^b^	0.07 ± 0.02 ^c^	0.85 ± 0.05 ^d^
Kaempferol	309.63 ± 0.56 ^a^	216.58 ± 0.51 ^b^	111.91 ± 0.24 ^c^	123.38 ± 0.27 ^d^	260.75 ± 0.16 ^e^	235.25 ± 0.52 ^f^
Luteolin	42.25 ± 0.23 ^a^	6.57 ± 0.10 ^b^	6.55 ± 0.12 ^b^	1.00 ± 0.08 ^c^	nd	3.54 ± 0.13 ^d^
Luteolin-7-O-glucosid	0.03 ± 0.02 ^a^	0.06 ± 0.03 ^a^	0.06 ± 0.04 ^a^	0.02 ± 0.01 ^a^	0.02 ± 0.01 ^a^	0.01 ± 0.01 ^a^
Myricetin	88.15 ± 0.34	nd	nd	nd	nd	nd
Naringenin	0.02 ± 0.0.01 ^b^	0.12 ± 0.02 ^b^	0.40 ± 0.06 ^a^	0.10 ± 0.02 ^b^	nd	nd
Quercetin	122.50 ± 0.18 ^a^	43.53 ± 0.11 ^b^	0.10 ± 0.04 ^c^	8.59 ± 0.09 ^d^	nd	0.60 ± 0.08 ^e^
Rutin	0.20 ± 0.04 ^d^	85.18 ± 0.25 ^a^	25.90 ± 0.23 ^b^	3.33 ± 0.10 ^c^	0.35 ± 0.05 ^d^	75.28 ± 0.22 ^e^
Trans-resveratrol	nd	nd	nd	nd	89.18 ± 0.25	nd
Carnosol	0.02 ± 0.01 ^a^	0.01 ± 0.01^a^	0.04 ± 0.02 ^a^	0.01 ± 0.01 ^a^	0.02 ± 0.01 ^a^	0.09 ± 0.04 ^a^
Salicin	33.65 ± 0.11 ^b^	23.30 ± 0.12 ^c^	71.67 ± 0.34 ^d^	nd	66.02 ± 0.33 ^e^	77.64 ± 0.27 ^a^

nd = not detected. dw = dry weight. Values are shown as mean ± standard deviation. Within the same row, different letters indicate significant differences (*p* < 0.05).

**Table 3:** Polyphenolic composition of the remaining solutions after the green synthesis of the BB-AgNPs.

Identified Compound, mg/mL (dw)	Sample					
	BB1	BB2	BB3	BB4	BB5	BB6
Caffeic acid	0.57 ± 0.02 ^a^	0.65 ± 0.04 ^a^	0.43 ± 0.05 ^b^	0.12 ± 0.02 ^c^	nd	0.65 ± 0.06 ^a^
Chlorogenic acid	nd	1.98 ± 0.07 ^a^	1.65 ± 0.06 ^b^	0.11 ± 0.02 ^c^	1.16 ± 0.08 ^d^	nd
*trans*-p-coumaric acid	13.70 ± 0.02 ^b^	22.87 ± 0.15 ^c^	8.16 ± 0.09 ^d^	3.47 ± 0.13 ^e^	0.04 ± 0.01 ^f^	28.21 ± 0.10 ^a^
Ellagic acid	11.74 ± 0.08 ^b^	nd	nd	23.54 ± 0.22 ^c^	61.38 ± 0.18 ^d^	65.56 ± 0.20 ^a^
Ferulic acid	nd	nd	nd	nd	nd	nd
Rosmarinic acid	nd	nd	nd	nd	nd	nd
Salicylic acid	1.23 ± 0.02 ^d^	12.71 ± 0.11 ^e^	4.82 ± 0.08 ^f^	13.98 ± 0.10 ^a^	0.32 ± 0.02 ^b^	1.02 ± 0.06 ^c^
Apigenin	nd	0.18 ± 0.06 ^b^	0.15 ± 0.04 ^b^	0.12 ± 0.02 ^b^	0.05 ± 0.02 ^c^	1.00 ± 0.09 ^a^
Chrysine	0.12 ± 0.05 ^b^	nd	nd	nd	0.51 ± 0.02 ^a^	0.15 ± 0.02 ^b^
Hyperoside	0.74 ± 0.04 ^a^	0.08 ± 0.02 ^b^	0.73 ± 0.05 ^a^	0.09 ± 0.02 ^b^	nd	0.25 ± 0.04 ^c^
Kaempferol	157.58 ± 0.41 ^a^	124.96 ± 0.32 ^b^	118.95 ± 0.21 ^c^	111.36 ± 0.10 ^d^	59.11 ± 0.16 ^e^	44.21 ± 0.18 ^f^
Luteolin	14.31 ± 0.11 ^a^	0.89 ± 0.09 ^b^	0.98 ± 0.12 ^b^	nd	nd	0.24 ± 0.04 ^c^
Luteolin-7-O-glucosid	nd	nd	nd	nd	nd	nd
Myricetin	36.15 ± 0.23	nd	nd	nd	nd	nd
Naringenin	nd	nd	0.12 ± 0.02	nd	nd	nd
Quercetin	61.78 ± 0.18 ^a^	18.37 ± 0.10 ^b^	nd	2.43 ± 0.02 ^c^	nd	nd
Rutin	nd	65.05 ± 0.14 ^a^	12.28 ± 0.11 ^b^	nd	nd	43.28 ± 0.10 ^c^
Trans-resveratrol	nd	nd	nd	nd	45.34 ± 0.17	nd
Carnosol	nd	nd	nd	nd	nd	nd
Salicin	21.32 ± 0.12 ^d^	11.76 ± 0.09 ^e^	52.08 ± 0.15 ^a^	nd	28.02 ± 0.13 ^b^	46.04 ± 0.17 ^c^

nd = not detected. dw = dry weight. The values from the table are represented as mean ± standard deviation. Within the same row, different letters indicate significant differences (*p* < 0.05).

**Table 4:** DLS data and zeta potential of BB-AgNP samples.

Sample	Average Hydrodynamic Diameter [nm]	PDI	Zeta Potential (mV)
BB1-AgNPs	48.3	0.390	+13.9
BB2-AgNPs	145.5	0.266	+12.0
BB3-AgNPs	119.6	0.365	+12.1
BB4-AgNPs	150.1	0.463	+13.3
BB5-AgNPs	65.4	0.432	+7.1
BB6-AgNPs	78.3	0.455	+8.3

PDI = Polydispersity index. All measurements were made in triplicate and average values are reported.

**Table 5:** Antioxidant activity of BB-AgNP samples.

Sample	DPPH Method (mg Trolox Equivalent/g)	ABTS Method (mg Trolox Equivalent/g)	FRAP Method (mmol/g Fe^II^)
BB1-AgNPs	20.54 ± 0.73 ^a^	56.64 ± 0.35 ^a^	12.34 ± 0.33 ^a^
BB2-AgNPs	12.46 ± 0.63 ^b^	39.56 ± 1.00 ^b^	7.38 ± 0.38 ^b^
BB3-AgNPs	10.71 ± 0.38 ^b^	36.82 ± 0.60 ^c^	6.35 ± 0.23 ^b^
BB4-AgNPs	6.71 ± 1.04 ^c^	29.81 ± 1.63 ^d^	3.97 ± 0.62 ^c^
BB5-AgNPs	9.36 ± 0.44 ^d^	38.10 ± 0.69 ^b^	5.54 ± 0.26 ^d^
BB6-AgNPs	13.41 ± 0.46 ^b^	42.82 ± 0.72 ^e^	8.17 ± 1.23 ^b^

Values are shown as mean ± standard deviation. Within the same column, different letters indicate significant differences (*p* < 0.05).

**Table 6:** Inhibition zones of AgNP samples against different microorganisms.

Bacterial Strain	Inhibition Diameters (mm)					
	BB1-AgNPs	BB2-AgNPs	BB3-AgNPs	BB4-AgNPs	BB5-AgNPs	BB6-AgNPs
Gram-Positive Bacteria						
*S. aureus*	13.65 ± 0.82 ^b e^	11.45 ± 0.87 ^b^	10.27 ± 0.58 ^c^	8.33 ± 1.08 ^d^	12.67 ± 0.98 ^e^	16.00 ± 1.08 ^a^
*B. cereus*	11.63 ± 0.63 ^c^	12.13 ± 0.98 ^c d^	12.33 ± 0.88 ^d^	10.00 ± 1.13 ^c^	18.71 ± 0.79 ^a^	16.82 ± 0.88 ^b^
*E. faecalis*	20.65 ± 0.95 ^a^	16.33 ± 0.85 ^b^	16.83 ± 0.58 ^b^	12.67 ± 0.89 ^c^	16.45 ± 1.31 ^b^	25.24 ± 0.72 ^b^
Gram-Negative Bacteria						
*E. coli*	19.56 ± 0.87 ^a^	19.31 ± 1.32 ^a^	17.17 ± 0.92 ^b^	14.27 ± 0.82 ^c^	16.50 ± 1.02 ^b^	18.17 ± 0.78 ^a^
*P. aeruginosa*	22.21 ± 1.06 ^a^	15.65 ± 0.65 ^b^	14.67 ± 0.78 ^b^	16.33 ± 0.72 ^b^	20.52 ± 0.83 ^c^	20.23 ± 0.72 ^c^
*S. enteritidis*	15.74 ± 1.19 ^a^	9.67 ± 0,75 ^b^	7.67 ± 0.59 ^c^	10.67 ± 0.59 ^b^	14.33 ± 0.86 ^a^	12.33 ± 1.12 ^b^
Yeast						
*C. albicans*	15.12 ± 0.83 ^a^	9.70 ± 0,73 ^c^	11.55 ± 1.12 ^b^	11.57 ± 0.81 ^b^	12.33 ± 0.78 ^b^	16.00 ± 0.85 ^a^

Values are shown as mean ± standard deviation. Within the same row, different letters indicate significant differences (*p* < 0.05).

**Table 7:** Minimum inhibitory concentration (MIC) of BB-AgNP samples against different microorganisms.

Bacterial Strain	MIC Value (µg/mL)					
	BB1-AgNPs	BB2-AgNPs	BB3-AgNPs	BB4-AgNPs	BB5-AgNPs	BB6-AgNPs
Gram-Positive Bacteria						
*S. aureus*	0.78	6.25	3.12	3.12	0.78	1.56
*B. cereus*	0.39	3.12	3.12	3.12	0.39	0.39
*E. faecalis*	0.39	3.12	1.56	1.56	1.56	0.39
Gram-Negative Bacteria						
*E. coli*	0.78	6.25	3.12	6.25	1.56	1.56
*P. aeruginosa*	0.39	1.56	1.56	0.78	0.39	0.39
*S. enteritidis*	0.39	12.5	6.25	6.25	1.56	0.39
Yeast						
*C. albicans*	0.39	3.12	3.12	0.78	0.39	0.78

## Data Availability

Not available.
